# Horse Civilisation in Shetland and Wales

**Published:** 1892-05-28

**Authors:** 


### EDITOR'S LETTER BOX.

[Our correspondents are reminded that prolixity of statement is a great bar to publication, and that brevity of style and conciseness of statement greatly facilitate early insertion.]

### HORSE CIVILISATION IN SHETLAND AND WALES.

Sir,—Don't think me a violent partizan, ready to run amuck against anyone who dares to say a harsh word about "Taffy," if I venture to call in question your observations under this heading in the "Annotations" (page 87), of which, by the way, I am a fairly regular and appreciative reader. I am a Welshman, and lived in Wales a good many years, and I must confess that the statement that "the Welshman is fiery, impulsive, turbulent, and defiant," fills me with amazement. Do I not know that the judges who have incited the Principality to "deliver the gaols," have found little or nothing to do, time after time, and have congratulated the inhabitants on the fact? Do I not know that in my native borough a prisoner to be tried at quarter sessions is a bird so rare that the Recorder's post is practically a sinecure, and that his stock of white gloves must be immense? We flattered ourselves in Wales that our quietness and orderliness, not to say our honesty, which is not now called in question, were so conspicuous that no section of the public could be oblivious of it, and now— ! I am afraid, too, that the theory as to the cause of the diversity in the manners of the Sheltie and the Welsh pony has no more solid foundation than many other pretty theories. The real cause is the difference in their upbringing. The Welsh ponies run wild on the hills, and acquire a deadly hatred of the dogs, by which they are frequently harried. This mode of life, though it may make them hardy and strong, does not make them gentle, and this is the whole "secret."—Yours faithfully,

T.

[The writer of the annotation thinks that "T." is probably right.—Ed.]

